# Multi-site musculoskeletal pain in Swedish police: associations with discomfort from wearing mandatory equipment and prolonged sitting

**DOI:** 10.1007/s00420-018-1292-9

**Published:** 2018-02-07

**Authors:** Louise Bæk Larsen, Elisabeth Elgmark Andersson, Roy Tranberg, Nerrolyn Ramstrand

**Affiliations:** 1 ADULT research group, Department of Rehabilitation, School of Health Sciences, Jönköping University, PO Box 1026, 551 11 Jönköping, Sweden; 2 CHILD research group, Department of Rehabilitation, School of Health and Welfare, Jönköping University, PO Box 1026, 551 11 Jönköping, Sweden; 3 Department of Orthopaedics, Institute of Clinical Sciences, University of Gothenburg, PO Sahlgrenska University Hospital, 413 45 Gothenburg, Sweden

**Keywords:** Body armour, Cross-sectional study, Duty belt, Law enforcement, Multi-site musculoskeletal pain

## Abstract

**Purpose:**

Musculoskeletal disorders are considered as a major issue affecting the health and well-being of active duty police. Discomfort from wearing mandatory equipment and sitting for long periods of time in fleet vehicles are workload factors linked to musculoskeletal disorders in police. This study aims to determine the prevalence of multi-site musculoskeletal pain among Swedish police and to explore the possible association to discomfort experience when wearing mandatory equipment and sitting for long periods in fleet vehicles.

**Methods:**

In this cross-sectional study responses from 4185 police were collected through a self-administered online survey including questions about physical work environment, mandatory equipment and musculoskeletal pain. Multi-site pain was determined through summing pain sites from four body regions. Binomial logistic regression was performed to explore the association between multi-site musculoskeletal pain: (1) discomfort from wearing mandatory equipment and (2) sitting for long periods in fleet vehicles.

**Results:**

The prevalence of multi-site musculoskeletal pain at least 1 day per week within the previous 3 months was 41.3%. A statistically significant association between discomfort from wearing mandatory equipment and multi-site musculoskeletal pain was found; duty belt [OR 5.42 (95% CI 4.56–6.43)] as well as body armour [OR 2.69 (95% CI 2.11–3.42)]. Sitting for long periods in fleet vehicles was not significantly associated to multi-site musculoskeletal pain.

**Conclusion:**

Multi-site musculoskeletal pain is a considerable problem among Swedish police and modifying mandatory equipment to decrease discomfort is suggested as a potential means of decreasing the musculoskeletal pain experienced by many police officers.

## Introduction

Musculoskeletal disorders are a major problem in the general working population, leading to sickness absence and limited work ability (Bevan et al. 2015; Hoy et al. 2010; Lidgren 2003; Monnier et al. 2015). Police are often considered as an occupational group with an increased risk of experiencing musculoskeletal disorders but large sample studies documenting prevalence are lacking. Musculoskeletal disorders among police have primarily been investigated through biomechanical studies, investigating different load carriage designs (Holmes et al. 2013; Larsen et al. 2016; Ramstrand et al. 2016) and through self-reports of pain (Nabeel et al. 2007) and discomfort (Filtness et al. 2014; Gyi and Porter 1998; Holmes et al. 2013; Ramstrand et al. 2016). Results to date have linked musculoskeletal disorders in police to; discomfort from wearing mandatory equipment such as duty belts and body armour (Filtness et al. 2014; Ramstrand and Larsen 2012; Ramstrand et al. 2016), prolonged periods of sitting in fleet vehicles (Filtness et al. 2014; Gyi and Porter 1998; Holmes et al. 2013; Ramstrand and Larsen 2012), and insufficient physical activity and fitness (Nabeel et al. 2007). The most frequently reported musculoskeletal disorder among police is low back pain but disorders in the neck, shoulders, arms and lower extremities are also common (Cho et al. 2014; Filtness et al. 2014; Gyi and Porter 1998; Holmes et al. 2013; Jahani et al. 2002; Ramstrand and Larsen 2012).

The Swedish police force has approximately 28,000 employees. Of these, more than one quarter work as uniformed active duty officers (Polisen 2015). Uniformed police are mandated to wear body armour and a duty belt containing essential equipment (weapon, OC spray, handcuffs, extra ammunition, torch, baton and radio) which must be worn at all times whilst on duty and adds an extra load of approximately 6–7 kg. Mandatory equipment can be considered as one of many workload factors that police are exposed to in their physical work environment. Shift work and sitting for long periods of time in fleet vehicles are also characteristic workload factors for this occupational group (Elgmark et al. 2013). To help individuals manage the physical nature of their work, Swedish police are permitted 1 h of physical exercise per week during paid working hours. The extent to which the above-mentioned workload factors are associated with the musculoskeletal disorders experienced by Swedish police is unknown.

As police are mandated to wear body armour and duty belts at all times, it is important to minimise any discomfort or adverse effect that may be associated with their use. Discomfort from wearing mandatory equipment has been reported among police during standing, walking and sitting in fleet vehicles (Filtness et al. 2014; Ramstrand and Larsen 2012; Ramstrand et al. 2016). Greatest discomfort from wearing mandatory police equipment has been reported in relation to the backrest bolster in standard police vehicles (Filtness et al. 2014). Filtness et al. (2014) indicated that the design of load carriage systems was a significant factor in relation to comfort, with a standard issued duty belt causing most discomfort when compared to a load-bearing vest.

Discomfort is a construct used in ergonomics to measure the subjective response to a specific task or environment (Annett 2002). In a prospective cohort study of healthy workers, Hamberg-van Reenen et al. (2008) demonstrated that work-related peak and cumulative musculoskeletal discomfort were predictors of future musculoskeletal pain in the lower back, neck and shoulders. The association between discomfort experienced by police when wearing mandatory equipment and musculoskeletal pain has not been investigated. Such data would allow police authorities to prioritise interventions aimed at reducing musculoskeletal pain in their employees and may provide valuable information for design of improved load carriage systems.

A common means of investigating musculoskeletal pain is to compare the number of pain sites reported by the individual. Multi-site musculoskeletal pain is usually defined as pain in two or more body sites at a certain point in time or during a defined retrospective period (Carnes et al. 2007; Neupane et al. 2013; Solidaki et al. 2010). Multi-site musculoskeletal pain is common in both the general (Carnes et al. 2007; Haukka et al. 2012) and working population (Miranda et al. 2010; Neupane et al. 2011) and considered to be more debilitating than single-site pain (Scudds and Robertson 2000). It has a high impact on disability (Picavet and Schouten 2003; Scudds and Robertson 2000), work ability (Miranda et al. 2010; Neupane et al. 2011, 2013; Phongamwong and Deema 2015) and sickness absence (de Fernandes and Burdorf 2016; Neupane et al. 2015). High occupational workloads, both physical and psychosocial, have been associated with multi-site musculoskeletal pain (Haukka et al. 2012; Neupane et al. 2016b; Sembajwe et al. 2013; Solidaki et al. 2010). Physical risk factors include; heavy lifting, high work pace with little recovery time, working in non-neutral body positions, repetitive movement patterns, whole-body or segmental vibration and static muscular load (Punnett and Wegman 2004). Psychosocial factors related to multi-site musculoskeletal pain include organisational and social context factors (Herin et al. 2014; Sembajwe et al. 2013). Age, gender (female), low levels of physical activity and obesity are also associated with multisite musculoskeletal pain (Haukka et al. 2012; Leveille et al. 2005; Neupane et al. 2016a; Pensola et al. 2016).

Multi-site musculoskeletal pain has been investigated in several occupational groups such as healthcare personal (Freimann et al. 2013; Neupane et al. 2016a; Sembajwe et al. 2013), kitchen workers (Haukka et al. 2012) and industrial workers (Neupane et al. 2013). To the authors’ knowledge, no studies of multi-site musculoskeletal pain have been conducted on police.

The primary aim of this study was to document the prevalence of multi-site musculoskeletal pain among Swedish police. A secondary aim was to explore the association between discomfort experienced from mandatory equipment, prolonged sitting in fleet vehicles and multi-site musculoskeletal pain. The researchers’ hypothesis for the secondary aim was that police who experience discomfort from wearing mandatory equipment and sit for long periods of time in fleet vehicles were more likely to report multi-site musculoskeletal pain.

## Method

### Study population and procedure

The sample of interest in the present study was police working as uniformed active duty officers. Information received by the researchers from each police municipality indicated that this group comprised of approximately 7400 individuals at the time of data collection.

Data were collected using a self-administered online survey. As it was not possible to identify police who only worked as uniformed active duty officers, the survey was distributed by internal e-mail to all employees of Swedish police (approximately 28,000), with a request that only active duty officers respond. Data were collected in February 2013 and, after 2 and 4 weeks, e-mail reminders were sent out to all employees. An initial control question regarding the respondents’ role in the police force ensured that data from only uniformed active duty officers were included in the analysis. A total of 4185 uniformed active duty officers responded to the survey, resulting in a response rate of 57%.

### Survey

The survey used in this study was primarily based upon questions from the Swedish Work Environment Survey (SWES). This survey was initially generated by Statistics Sweden (SCB) and The Swedish Work Environment Authority who collect and report SWES data biennially on a sample representing the Swedish working population (Arbetsmiljöverket 2016). The SWES consists of questions related to the respondent’s physical and psychosocial work environment. In the present study, the focus was on questions related to the physical work environment.

In addition to the SWES, questions were also included in the survey to gain a deeper understanding of the demographics of the sample, use of mandatory equipment and occupation-specific work environment issues. The additional questions were developed by a working group consisting of the authors and two employees of The Swedish National Police force. Pilot tests were conducted prior to data collection by distributing the survey to a group of 30 police and conducting focus group discussions with representatives from this group. The first focus group was conducted with participants working in a larger city (*n* = 5) and the second with participants working in a smaller city (*n* = 4). The survey was discussed and subsequently modified for the purpose of clarification. The final survey included 146 questions.

### Outcome

Multisite musculoskeletal pain was the main outcome variable and was recorded separately for four body regions, including (1) the upper back or neck, (2) lower back, (3) shoulders or arms and (4) hips, legs, knees or feet. Each item was assessed with the question; “During the last 3 months have you, after work, experienced pain in [region]…?” Response alternatives were given on a 5-point scale (1 = not at all/seldom, 2 = a few days per month, 3 = 1 day per week, 4 = a few days per week or 5 = every day). Scores for each body region were dichotomised into two new categories 0 = no pain/seldom or a few days per month and 1 = pain at least 1 day per week or more. Using these data, multi-site musculoskeletal pain was subsequently determined as pain in two or more body regions.

### Exposure

#### Duty belt and body armour

Discomfort from wearing a duty belt was assessed by two items. The questions were phrased as follows: “Do you experience physical discomfort from wearing a duty belt while walking and standing?” and “Do you experience physical discomfort from wearing duty belt while seated in fleet vehicle?” A 4-point response scale was used (1 = not at all, 2 = mostly not, 3 = mostly or 4 = always). Scores for both items were dichotomised at the median 3 (less than median: 0 = no experience of discomfort; more than median: 1 = experience of discomfort). Discomfort from wearing duty belt was subsequently classified as a response of 1 on both items. A similar procedure was performed for discomfort experienced from wearing body armour.

#### Sitting in fleet vehicles

Time spent sitting in fleet vehicles was assessed by one item. The question was phrased as follows: “What percentage of an average work shift do you spend sitting in a fleet vehicle as driver or passenger?” Response alternatives were (1 = never, 2 = less than 25%, 3 = 25–50%, 4 = 50–75% or 5 = 75% or more).

### Covariates

#### Physical workload factors

Physical workload factors that could adversely affect police were measured with five items and the questions were phrased as follows: “Does your work sometimes require that you strain yourself physically?”, “Do you strain yourself to a degree that requires you to breath faster?”, “Do you work in a forward bent position without support from hands or arms?”, “Do you work in awkward /rotated postures?” and “Do you work with your hands above shoulder level”. A 6-point response scale was used (1 = not at all, 2 = 1/10 of the time, 3 = 1/4, 4 = half the time, 5 = 3/4 of the time or 6 = almost all the time). Summing of all five items created a composite variable ranging from 5 to 30 which was categorised into three groups representing low (5–13), medium (14–22) and high (23–30) physical workload exposure (Neupane et al. 2016a). Cronbach’s alpha for the composite variable was 0.79.

#### Psychosocial factors

Psychosocial factors were addressed using indices representing job demands, job control and job support (Elgmark Andersson et al. 2017; Arbetsmiljöverket 2016). The index representing job demands was based upon four items related to the possibility to take rests during working hours, overtime, stressful working conditions, and feeling like they have too much to do at work. The index for job control was also generated from four items and addressed the possibility to influence work in terms of planning, methods, pace and order of events, as well as the overall feeling of being able to influence one’s own work. All items included in the job demand and control indices were dichotomised according to procedures documented by the Swedish Work Environment Authority (SWEA). Scores for each question were summed to give a total score between 0 and 4. Indices for both job demands and job control were subsequently categorised as low (< 2) or high (≥ 2). The index representing job support was created from six items in the SWES. These items have been described in detail by Elgmark Andersson et al. (2017). Each item for job support were dichotomised and summed into a new variable ranging from 0 to 6. The support index was then divided into low support (< 3) and high support (≥ 3).

#### Physical exercise

Physical exercise was assessed with one question, “How many hours per week do you exercise?” A 4-point response scale was used (1 = not at all, 2 = 1–2 h, 3 = 3–4 h or 4 = more than 4 h). Scores were dichotomised at the median 3 (less than median: 0 = 2 h or less per week; more than median: 1 = more than 2 h per week).

#### Age and gender

Age and gender were included as covariates in the analysis. The question about age had seven response categories (20–24, 25–29, 30–34, 35–39, 40–44, 45–49 or 50+).

## Statistical analysis

Prevalence rates for musculoskeletal pain, categorised as no pain, single-site pain and multi-site pain were calculated for the total sample (see Table 1). Descriptive statistics for exposure variables and covariates were calculated for the total sample and stratified into (1) no pain or single-site and (2) multi-site (see Table 2).

**Table 1:** Prevalence of multisite musculoskeletal pain at least one time per week within the last 3 months

	Total	
	*N* = 4185	Column %
No pain	1561	37.3
Single-site pain	825	19.7
Multi-site pain	1728	41.3
Missing	71	1.7

**Table 2:** Descriptive statistics stratified into no pain or single-site pain and multi-site pain

	Total		No pain or single-site pain		Multi-site pain	
	*N* = 4114	Colum %	*N* = 2386	Row %	*N* = 1728	Row %
Gender						
Men	3028	73.6	1805	59.6	1223	40.4
Women	1063	25.8	571	53.7	492	46.3
Missing	23	0.6	10		13	
Age group (years)						
20–24	32	0.8	14	43.8	18	56.3
25–29	817	19.9	470	57.5	347	42.5
30–34	1204	29.3	695	57.7	509	42.3
35–39	825	20.1	480	58.2	345	41.8
40–44	521	12.7	287	55.1	234	44.9
45–49	243	5.9	143	58.8	100	41.2
50+	449	10.9	279	62.1	170	37.9
Missing	23	0.6	18		5	
Physical exercise/week						
< 2 h	1240	30.1	693	55.9	547	44.1
> 2 h	2872	69.8	1693	58.9	1179	41.1
Missing	2	0.0			2	
Duty belt						
No discomfort	2839	69.0	2018	71.1	821	28.9
Discomfort	1215	29.5	325	26.7	890	73.3
Missing	60	1.5	43		17	
Body armour						
No discomfort	3472	84.4	2198	63.3	1274	36.7
Discomfort	584	14.2	159	27.2	425	72.8
Missing	58	1.4	29		29	
Exposure to physical workload factors						
Low	3090	75.1	1935	62.6	1155	37.4
Medium	882	21.4	370	42.0	512	58.0
High	47	1.1	11	23.4	36	76.6
Missing	95	2.3	70		25	
Job demands						
Low	2153	52.3	1407	65.4	746	34.6
High	1894	46.0	934	49.3	960	50.7
Missing	67	1.6	45		22	
Job control						
Low	2980	72.4	1656	55.6	1324	44.4
High	1056	25.7	677	64.1	379	35.9
Missing	78	1.9	53		25	
Social support						
Low	457	11.1	222	48.6	235	51.4
High	3545	86.2	2096	59.1	1449	40.9
Missing	112	2.7	68		44	
Sitting in vehicle % of shift						
Not at all	11	0.3	6	54.5	5	45.5
< 25%	379	9.2	237	62.5	142	37.5
25–50%	1650	40.1	994	60.2	656	39.8
50–75%	1715	41.7	968	56.4	747	43.6
> 75%	348	8.5	174	50.0	174	50.0
Missing	11	0.3	7		4	

Row% = sum of no pain or single-site pain + multi-site pain

A binominal logistic regression was performed to ascertain the effects of exposure variable and covariates on multi-site pain (Table 3). Odds ratios were calculated as measures of association. Tests for the assumptions of binominal logistic regression were performed by investigating for multicollinearity and outliers. No multicollinearity between the independent variables was found. There were 20 studentized residuals with values ranging from − 2.55 to − 3.51. These were removed from the analysis. Removing the outliers did not change the main result. Exposure variables and covariates were inserted to the regression model in three steps. The first model included variables of specific interest to the aim of this study, including discomfort from duty belt and body armour and time spent sitting in a fleet vehicle (Model I). The second model included model I + exposure to physical workload factors and psychosocial factors (Model II). The third model included model II + the covariates physical exercise, age and sex (Model III). All statistical analyses were carried out using IBM SPSS Statistics 21 (IBM Corp, Armonk, NY, USA).

**Table 3:** Odds ratios (OR) with 95% confidence intervals for multi-site musculoskeletal pain at least one time per week within the last 3 months

	OR	95% CI		OR	95% CI		OR	95% CI	
		Lower	Upper		Lower	Upper		Lower	Upper
	Model I			Model II			Model III		
Duty belt									
No discomfort	1			1			1		
Discomfort	5.81**	4.91	6.86	5.42**	4.57	6.42	5.42**	4.56	6.43
Body armour									
No discomfort	1			1			1		
Discomfort	2.85**	2.25	3.61	2.73**	2.15	3.48	2.69**	2.11	3.42
Time of shift sitting in vehicle (%)									
< 25%	1			1			1		
25–50%	0.94	0.72	1.22	0.93	0.71	1.22	0.97	0.74	1.28
50–75%	1.07	0.82	1.39	1.06	0.81	1.39	1.11	0.84	1.47
> 75%	1.13	0.80	1.59	1.05	0.74	1.50	1.10	0.77	1.57
Exposure to physical workload factors									
Low	**–**	**–**	**–**	1			1		
Medium	**–**	**–**	**–**	1.91**	1.60	2.28	1.95**	1.63	2.34
High	**–**	**–**	**–**	3.42**	1.61	7.26	3.50**	1.64	7.43
Job demands									
Low	**–**	**-**	**-**	1			1		
High	**–**	**–**	**–**	1.46**	1.25	1.70	1.46**	1.25	1.71
Job control									
Low	**–**	**–**	**–**	1			1		
High	**–**	**–**	**–**	1.02	0.85	1.21	1.00	0.83	1.19
Social support									
Low	**–**	**–**	**–**	1			1		
High	**–**	**–**	**–**	0.82	0.65	1.03	0.85	0.67	1.07
Physical exercise per week									
< 2 h	**–**	**–**	**–**	**–**	**–**	**–**	1		
> 2 h	**–**	**–**	**–**	**–**	**–**	**–**	0.82*	0.70	0.97
Age									
25–29 years	**–**	**–**	**–**	**–**	**–**	**–**	1		
30–34 years	**–**	**–**	**–**	**–**	**–**	**–**	1.05	0.85	1.30
35–39 years	**–**	**–**	**–**	**–**	**–**	**–**	1.06	0.84	1.35
40–44 years	**–**	**–**	**–**	**–**	**–**	**–**	1.29	0.98	1.69
45–49 years	**–**	**–**	**–**	**–**	**–**	**–**	1.18	0.83	1.68
> 50 years	**–**	**–**	**–**	**–**	**–**	**–**	1.12	0.82	1.52
Sex									
Men	**–**	**–**	**–**	**–**	**–**	**–**	1		
Women	**–**	**–**	**–**	**–**	**–**	**–**	1.17	0.98	1.39

Model I. Duty belt, body armour and sitting in vehicle

Model II. Model I + exposure to physical work load factors and psychosocial factors

Model III. Model II + physical exercise, age and sex

Significant results are shown in bold. *P < 0.05 and **P < 0.001

## Result

A total of 4114 participants of the original 4185 who responded to the survey were included in the analysis. Those not included (*n* = 71) did not answer the questions related to musculoskeletal pain. Prevalence of musculoskeletal pain in the four body sites were: upper back or neck (33.7%), lower back (43.2%), shoulders or arms (25.5%) and hips, legs, knees or feet (38.0%). Multi-site musculoskeletal pain was reported by 41.3% of the participants, see Table 1.

Descriptive statistics related to exposure variables and covariates are presented in Table 2. The variables are presented for the total sample and stratified into (a) no pain or single-site pain and (b) multi-site pain. Male police officers represented three quarters of the participants and the majority of participants were 25–39 years of age. Physical exercise more than 2 h per week was undertaken by the majority of participants and over 80% reported sitting 25–75% of their average work shift in police vehicles. Multi-site musculoskeletal pain was reported by 70% of those participants experiencing discomfort from wearing duty belt and body armour.

Results of binominal logistic regression showed that discomfort experienced from wearing a duty belt and body armour was significantly related to multi-site musculoskeletal pain in all three models, see Table 3. For models I–III, odds ratios for the independent variable discomfort from duty belt were twice that recorded for discomfort from body armour. Odds ratios for discomfort from duty belt ranged from 5.81 to 5.42 while odds ratios for discomfort from body armour ranged from 2.85 to 2.69.

## Discussion

This study investigated the prevalence of multi-site musculoskeletal pain among Swedish police, as well as the association between discomfort experienced from wearing mandatory equipment, sitting for long periods in fleet vehicles and multi-site musculoskeletal pain. Multi-site musculoskeletal pain, experienced at least 1 day per week in the 3-month period prior to answering the survey, was reported more frequently than no pain and twice as much as single-site pain. Discomfort experienced from wearing mandatory equipment was significantly associated with multi-site musculoskeletal pain. Discomfort experienced from wearing a duty belt had the strongest overall association with an odds ratio of almost six.

41% of participants reported that they experience multi-site musculoskeletal pain, a figure that is consistent with previous research involving the healthcare sector and industrial workers (Freimann et al. 2013; Neupane et al. 2011, 2016a; Sembajwe et al. 2013). These previously studied occupational groups have similarities with police regarding shift work and exposure to physical workload factors such as heavy lifting and working in twisted and awkward postures. While period prevalence in previous studies range from 1 week to 6 months, the results are consistent in showing that multi-site musculoskeletal pain is more common than single-site pain.

Lower back pain was the most frequently reported pain site among participants. A result which confirms previously reported data related to police (Brown et al. 1998; Cho et al. 2014; Gyi and Porter 1998; Jahani et al. 2002; Ramstrand and Larsen 2012). Jahani et al. (2002) and Cho et al. (2014) found the prevalence of lower back pain among police to be 43.6 and 41.1%, respectively, which is similar to findings from this study (43.2%). According to SWEA, the prevalence of lower back pain in the Swedish general working population was 32% during the same period that data for the present study was collected (Arbetsmiljöverket 2014). While SWEA indicates that there is substantial variation in prevalence of lower back pain for different occupational groups, the result for Swedish police is considered high compared to the general working population. Lower back pain is known to impact the life of the individual in terms of activity limitations and work absenteeism and represents a substantial financial burden for society (Hoy et al. 2010). As such, reducing musculoskeletal pain should be prioritised within the Swedish police.

The association between discomfort experienced from wearing duty belt, body armour and multi-site musculoskeletal pain supports suggestions from previous authors of a relationship between carrying mandatory police equipment and lower back pain (Brown et al. 1998; Burton et al. 1996; Filtness et al. 2014; Holmes et al. 2013). No prior study has investigated multi-site musculoskeletal pain among police, and therefore, comparisons to previous work is somewhat limited. While discomfort from wearing duty belt and body armour were both strongly associated to multi-site musculoskeletal pain, the duty belt was found to have the greatest association. There are a number of biomechanical factors that may contribute to musculoskeletal problems in Swedish police. Previous work has demonstrated that police duty belts cause limitations in range of motion of the right hip and an abducted position of the arms during normal walking (Larsen et al. 2016; Ramstrand et al. 2016). The restriction in range of motion for the right hip is likely due to the position of the weapon whereas walking with abducted arms could potentially be due to the increased width around the pelvis caused by the duty belt. Walking with abducted arms for longer periods of time would put extra strain on shoulders and upper back/neck suggesting that the duty belt can affect more than one body region and be associated to multi-site musculoskeletal pain. Holmes et al. (2013) studied perceived discomfort from wearing duty belt during simulated driving. Results showed that female police experienced more discomfort in general and that lower back, pelvis and right thigh and buttocks were body regions recording the most discomfort for both males and females. Wearing body armour has been found to decrease range of motion in the trunk during walking (Larsen et al. 2016) and Burton et al. (1996) found an increased risk for first onset of lower back pain related to wearing body armour among a group of police in Northern Ireland. However, the weight of body armour presented by Burton 2 decades ago was four times that of those worn by Swedish police at the time of data collection.

Sitting for long periods of time in fleet vehicles was not significantly associated with multi-site musculoskeletal pain among police. This was an unexpected result as earlier studies have identified sitting in vehicles as a problem for police (Burton et al. 1996; Filtness et al. 2014; Gyi and Porter 1998; Holmes et al. 2013) as well as other occupational drivers (Mozafari et al. 2015; Porter and Gyi 2002). It is likely that the observed differences are due to the fact that the present study focused upon multi-site pain, whereas previous studies have investigated only single pain sites and in particular lower back pain.

This study represents the largest study sample of investigating musculoskeletal pain in police. The response rate of 57% is considered very good for an online survey, which are known to have a lower response rate than paper-based surveys (Nulty 2008). As it was not possible to perform any analysis of participants who chose not to respond to the survey, we cannot comment on any potential bias related to the sample included in this analysis. In addition, the cross-sectional design used in this study does not allow us to draw conclusions about causality. It must also be recognised that the main independent variable, discomfort, and the dependent variable, pain (multi-site pain), might be viewed as two related concepts per se. To disentangle the relationship between wearing mandatory equipment and pain, a comparison group of police not wearing a duty belt and body armour would have been preferable. Given that use of a duty belt and body armour is mandated by police authorities, such a comparison was ethically not possible. This study does not take into account possible interaction effects between the independent variables.

Multi-site musculoskeletal pain is a frequently reported problem among Swedish uniformed active duty officers. Results of this study have identified discomfort experienced from wearing mandatory equipment as an important variable when addressing the high prevalence of multi-site musculoskeletal pain among Swedish police. Priority should be given to improving comfort when wearing duty belts.
